# Diagnostic performance of axillary-vein distensibility index (AXV-DI) versus inferior-vena-cava distensibility index (IVC-DI) for predicting fluid responsiveness in patients with septic shock

**DOI:** 10.3389/fmed.2026.1926984

**Published:** 2026-10-28

**Authors:** Xiaoyun Zou, Xianzhou Quan, Haien Yu, Fei Guo, Jianguo Zhang, Peng Shen

**Affiliations:** 1 Department of Critical Care Medicine, Women and Children’s Hospital, Qingdao University, Qingdao, China; 2 Department of Critical Care Medicine, Peking University People’s Hospital, Qingdao, China; 3 Department of Critical Care Medicine, Chengyang District People’s Hospital, Qingdao, China; 4 Department of Critical Care Medicine, Peking University People’s Hospital, Beijing, China

**Keywords:** axillary vein, inferior vena cava, mechanical ventilation, septic shock, volumetric responsiveness

## Abstract

**Background:**

Septic shock relies on early goal-directed fluid resuscitation. Inferior vena cava (IVC) ultrasound is standard for volume evaluation, yet the IVC is frequently affected by abdominal distension, emphysema or high intra-abdominal pressure. This study aimed to compare the diagnostic performance of axillary vein distensibility index (AXV-DI) and inferior vena cava distensibility index (IVC-DI) for assessing fluid responsiveness in mechanically ventilated patients with septic shock in the intensive care unit.

**Materials and methods:**

This was a single-center prospective observational study that enrolled 95 mechanically ventilated patients with septic shock in the ICU. A standardized fluid challenge with 500 mL of Lactated Ringer's solution infused intravenously over 15 min was performed. The primary outcome was the comparison of the diagnostic performance (area under the receiver operating characteristic curve) of AXV-DI vs. IVC-DI for predicting fluid responsiveness. The secondary outcome was the changes in hemodynamic and ultrasonic parameters before and after standardized fluid challenge in responder and non-responder groups.

**Results:**

Eighty-six patients were analyzed (60 responders, 26 non-responders). For the primary outcome, the ROC AUC values for predicting fluid responsiveness were 0.93 for IVC-DI and 0.80 for AXV-DI. The DeLong test confirmed that IVC-DI exhibited significantly superior diagnostic performance compared with AXV-DI (*P* = 0.009). For the secondary outcome, responders presented more significant changes in LVOT-VTI and venous distensibility indices after fluid resuscitation compared with non-responders. Baseline hemodynamic and ultrasonic parameters were significantly different between the two groups.

**Conclusions:**

Both AXV-DI and IVC-DI can predict fluid responsiveness in patients with septic shock, although AXV-DI exhibits lower diagnostic performance than IVC-DI.

## 1 Introduction

Sepsis and septic shock are major healthcare problems, impacting millions of people around the world each year and killing between one in three and one in six of those it affects (1). Fluid therapy is designed to increase stroke volume (SV) and cardiac output (CO), thereby optimizing systemic blood flow and tissue perfusion (2). Insufficient fluid resuscitation may lead to progressive tissue hypoperfusion and organ dysfunction, while excess fluids could induce detrimental fluid overload (3, 4). Multiple clinical practice guidelines have consistently demonstrated that patients with sepsis exhibit significant interindividual variability in fluid volume requirements, and the 2026 Surviving Sepsis Campaign (SSC) guidelines also reiterate the recommendation for frequent clinical reassessment of patients to strictly mitigate the risks of inadequate fluid resuscitation or fluid overload (5, 6). Numerous parameters are used to predict fluid responsiveness. The conventional methods for postoperative volume assessment involve observing skin elasticity, urine volume, blood pressure, and heart rate (HR). While these methods are convenient, rapid, and do not require special operations, their accuracy cannot be precisely quantified. Traditional physical examination signs and static hemodynamic parameters lack sufficient accuracy for predicting fluid responsiveness, while invasive monitoring is limited by operational complexity and clinical accessibility (7). Ultrasonography is widely utilised to measure the respiratory variability of the inferior vena cava (IVC) for predicting fluid responsiveness owing to its simplicity, non-invasiveness, and reproducibility. A 2025 meta-analysis demonstrated that the inferior vena cava distensibility index (IVC-DI) yielded high diagnostic value (AUC = 0.88) among mechanically ventilated patients (8). However, IVC visualization is frequently impaired by abdominal distension, subcutaneous emphysema, and elevated intra-abdominal pressure, which substantially restricts its clinical applicability (9). Physiologically, the proximal axillary vein is largely unaffected by elevated intra-abdominal pressure, which may confer a potential physiological advantage. This study aimed to comprehensively compare the diagnostic performance of AXV-DI and IVC-DI in mechanically ventilated patients with septic shock who had adequate IVC ultrasound images.

Previous studies have shown that AXV diameter and its respiratory-induced variation are closely associated with central venous pressure and fluid status (10, 11). Observational studies have reported moderate diagnostic performance of respiratory-variation-derived AXV-related indices in hemodynamic assessment, and the infraclavicular axillary vein collapsibility index (AXV-CI) satisfactorily predicted anesthesia-induced hypotension in elderly gastrointestinal-surgery patients (10). Accordingly, this study aimed to comprehensively compare the diagnostic performance of AXV-DI and IVC-DI in mechanically ventilated patients with septic shock.

## 2 Materials and methods

This study was derived from an ethically-approved project entitled “Application of bedside ultrasound technology in central vein catheterization and hemodynamic monitoring–simulation and practice of critical care ultrasound training”. The parent project was approved by the Ethical Review Committee of Peking University People's Hospital (Approval No. 2020PHB118-01). This prospective observational study was approved by the Ethics Committee of Peking University People's Hospital. All patient enrollment and data collection were completed within the 6-year validity period of the ethical approval. Written informed consent was obtained from patients or their legal surrogates prior to enrollment. All procedures were performed in accordance with the institutional ethical standards and the principles of the 1964 Declaration of Helsinki and its subsequent amendments. Since this was a non-interventional observational study, clinical trial registration was not required. All statistical analyses were strictly conducted following the pre-specified analysis protocol.

### 2.1 Study design

This was a single-center, prospective observational study. All ultrasonographic and hemodynamic data were prospectively collected at the bedside. Participants were retrospectively screened from the prospectively enrolled dataset according to predefined inclusion and exclusion criteria. All research objectives and statistical analysis plans were finalized prior to data analysis, with no *post-hoc* modifications throughout the study.

### 2.2 Patients

Patient recruitment and data collection were performed in the 15-bed intensive care unit (ICU) of Peking University People's Hospital Qingdao Hospital. Eligible patients were diagnosed with septic shock according to the Sepsis-3 criteria (12).

Inclusion criteria were as follows: (1) Age ≥18 years; (2) Patients transferred from the emergency department or general ward, fulfilling the diagnostic criteria for septic shock according to the Sepsis-3 criteria, receiving controlled mechanical ventilation, and requiring intravenous fluid-challenge resuscitation based on clinical assessment (12).

Exclusion criteria were as follows: (1) Cardiac arrhythmia; (2) Refractory ascites; (3) Pre-existing severe valvular heart disease and/or intracardiac shunt; (4) Contraindications to sedatives and/or neuromuscular blocking agents; (5) Pneumothorax, massive pleural effusion, or superior vena cava syndrome; (6) Pregnancy; (7) Poor ultrasonographic visualization of the inferior vena cava caused by severe obesity, intra-abdominal hypertension (pressureintra-abdominal pressure ≥12 mmHg) (13), large abdominal space-occupying lesions, or severe intestinal distension; (8) Suspected pulmonary embolism; (9) Failure to receive internal jugular vein catheterization for central venous pressure (CVP) monitoring; (10) Patient refusal to participate.

All patients were given temporary sedation and neuromuscular blockade, and received fully controlled mechanical ventilation using the Mindray SV300 ventilator (Mindray, Shenzhen, China). No spontaneous breathing was detected on ventilator waveforms, confirming that respiratory-related cyclic changes in venous diameter were exclusively induced by positive-pressure ventilation. Ventilation was set to volume-controlled mode: positive end-expiratory pressure (PEEP) 5 cm H_2_O, tidal volume 8 mL/kg predicted body weight, respiratory rate 15 breaths/min, and plateau pressure kept below 30 cm H_2_O (14).

### 2.3 Measurement

General baseline data were collected for all patients, including age, gender, height, weight, body-mass index (BMI), Acute Physiology and Chronic Health Evaluation II (APACHE-II) score, and Sequential Organ Failure Assessment (SOFA) score. The infusion rate of norepinephrine (μg·kg⁻^1^·min⁻^1^) at the time of admission from the emergency department to the ICU was recorded. A central venous catheter was inserted via the right internal jugular vein for CVP monitoring, with the zero reference point set at the level of the fourth intercostal space on the right mid-axillary line. Radial artery puncture was performed to monitor arterial pressure, and MAP and HR were documented. Arterial blood lactate levels were recorded on admission based on arterial blood gas analysis.

Upon admission to the intensive care unit (ICU), bedside ultrasonography was immediately performed to measure the inferior vena cava distensibility index (IVC-DI) and axillary vein distensibility index (AXV-DI). Meanwhile, the left ventricular outflow tract velocity-time integral (LVOT-VTI) was measured. Subsequently, a standardized fluid challenge was administered: 500 mL of Lactated Ringer's solution was infused intravenously over 15 min (14). After fluid expansion, changes in hemodynamic parameters (HR, CVP, MAP) and ultrasonic indices (IVC-DI, AXV-DI, LVOT-VTI) were recorded again.

Bedside ultrasonography was performed using a Mindray ME7 Doppler ultrasound system. All transthoracic ultrasound measurements were conducted as follows.

The LVOT-VTI was acquired via pulsed-wave Doppler at the apical five-chamber view using a 3.5 MHz transducer. Each measurement was repeated three times and the average value was calculated. These measurements were performed by an independent intensivist, who was blinded to all venous ultrasound results of AXV-DI and IVC-DI.

For IVC assessment: A 3.5 MHz low-frequency probe was applied in B-mode at the subcostal window. The IVC diameter was measured at approximately 1 cm distal to the hepatic vein orifice. The maximum (dIVCmax) and minimum (dIVCmin) IVC diameters within one respiratory cycle were recorded. The IVC-DI was calculated according to the formula: IVC-DI = [(dIVCmax − dIVCmin)/dIVCmin] × 100% (15).

For AXV assessment: With the patient in the supine position, a 7.5 MHz linear array probe was used to locate the right axillary vein. The third segment of the axillary vein was selected. Scanning was performed gradually inward and outward below the midpoint of the clavicle perpendicular to the clavicular direction. The probe was then placed at the superior border of the pectoralis minor muscle and the lateral margin of the first rib to obtain the optimal cross-sectional view of the axillary vein. Color Doppler or spectral Doppler was applied to identify and distinguish the axillary vein from the axillary artery in the short-axis view (10). The probe was rotated to be parallel to the clavicle, and the subclavian long-axis view was used to measure the maximal (dAXVmax) and minimal (dAXVmin) diameters of the axillary vein during one respiratory cycle. The AXV-DI was calculated as follows: AXV-DI = [(dAXVmax − dAXVmin)/dAXVmin] × 100%. During measurement, the patient's upper extremity was kept beside the body in neutral position without shoulder abduction or rotation. Minimal probe pressure was applied to prevent extrinsic compression of the axillary vein. Images were acquired at end-expiration under mechanical ventilation. Only images with complete, clearly visualized vessel lumen without deformation were included (16). Consistent with LVOT-VTI, AXV-DI and IVC-DI were measured three times offline, and the average value was adopted for analysis.

Ventilator settings and intravenous medications remained unchanged throughout the study period. All index-test ultrasound measurements, including AXV-DI and IVC-DI acquisition and calculation, were performed by a different physician with 5 years of experience in critical care ultrasonography. This operator was blinded to LVOT-VTI values and the final fluid-responsiveness classification.

Taking a 15% increase in LVOT-VTI as the cutoff for fluid responsiveness, patients were classified into two groups: the responder group (LVOT-VTI increase ≥15%) and the non-responder group (LVOT-VTI increase <15%) (17, 18).

### 2.4 Study outcomes

The primary outcome of this study was to compare the diagnostic performance of the axillary vein distensibility index (AXV-DI) and inferior vena cava distensibility index (IVC-DI) for predicting fluid responsiveness in mechanically ventilated patients with septic shock. The diagnostic accuracy of the two indices was evaluated using the area under the receiver operating characteristic curve (AUC). The DeLong test was used to compare AUC values between AXV-DI and IVC-DI, and the Youden index was applied to determine the optimal cut-off value, sensitivity, and specificity of each index. The secondary outcome was to analyze the alterations in AXV-DI, IVC-DI, and other hemodynamic parameters before and after standardized fluid challenge in fluid responder and non-responder groups. Analyses for the secondary outcome were considered exploratory, and no multiplicity adjustment was applied; *P*-values are presented for descriptive purposes.

### 2.5 Statistical analysis

All continuous variables were tested for normality via the Shapiro–Wilk test, which confirmed non-normal distributions for all indicators (*P* < 0.05). Thus, continuous variables were reported as median (interquartile range, IQR).

Non-parametric statistical tests were thus used for all continuous variable analyses: the Mann–Whitney *U* test for intergroup comparisons (indicators between fluid responders and non-responders at the same time point), and the Wilcoxon signed-rank test for intragroup comparisons (pre- and post-resuscitation indicators within each group). The *Z*-value was reported as the test statistic for both non-parametric approaches.

Categorical variables were presented as *n* (%), and intergroup comparisons were conducted using the Pearson *χ*^2^ test, with *χ*^2^ values and degrees of freedom (df) reported. Fisher's exact test was applied for primary infection site comparisons when more than 20% of table cells had expected frequencies <5. The 95% confidence intervals for sensitivity and specificity at the pre-specified optimal cut-off point derived from the full original cohort were computed via the bootstrap method with 2000 stratified resamples. The cut-off value was kept fixed across all bootstrap replicates. The De-Long-based bootstrap procedure was applied to compare the AUCs of the two ROC curves. For secondary-outcome-related comparisons, including baseline inter-group comparisons, intra-group pre-post comparisons after fluid challenge, and change-score Δ analyses between responders and non-responders, these analyses were considered exploratory. Therefore, no multiplicity adjustment was performed, and *P*-values for exploratory analyses are reported for descriptive purposes only.

All statistical tests were two-sided, and *P* < 0.05 was considered statistically significant. Core statistical calculations (*Z*-values, *χ*^2^ values, df, *P*-values) were performed using SPSS version 27.0 (IBM Corporation, Armonk, New York, USA). All figures and related analyses, including receiver operating characteristic (ROC) curves, boxplots of IVC-DI/AXV-DI across responder subgroups, line charts of VTI before and after fluid challenge, were visualized in R software (version 4.5.3; R Foundation for Statistical Computing, Vienna, Austria). The ggplot2, pROC, and ggpubr packages were used for plotting.

## 3 Results

3.1 A total of 95 patients diagnosed with septic shock were screened for eligibility at the time of enrolment. Four patients were excluded due to poor ultrasonographic visualization of the inferior vena cava, and another 5 were excluded because the axillary vein could not be measured secondary to anatomical variations. Among the 86 enrolled patients, the median age was 53 years, and 65.1 % of patients were male. The most common site of infection was the respiratory tract. All 86 patients were further divided into the fluid non-responder group (*n* = 26) and the fluid responder group (*n* = 60) according to fluid-responsiveness, and included in the final diagnostic-accuracy analysis. The recruitment flow is illustrated in Figure 1.

**Figure 1:**
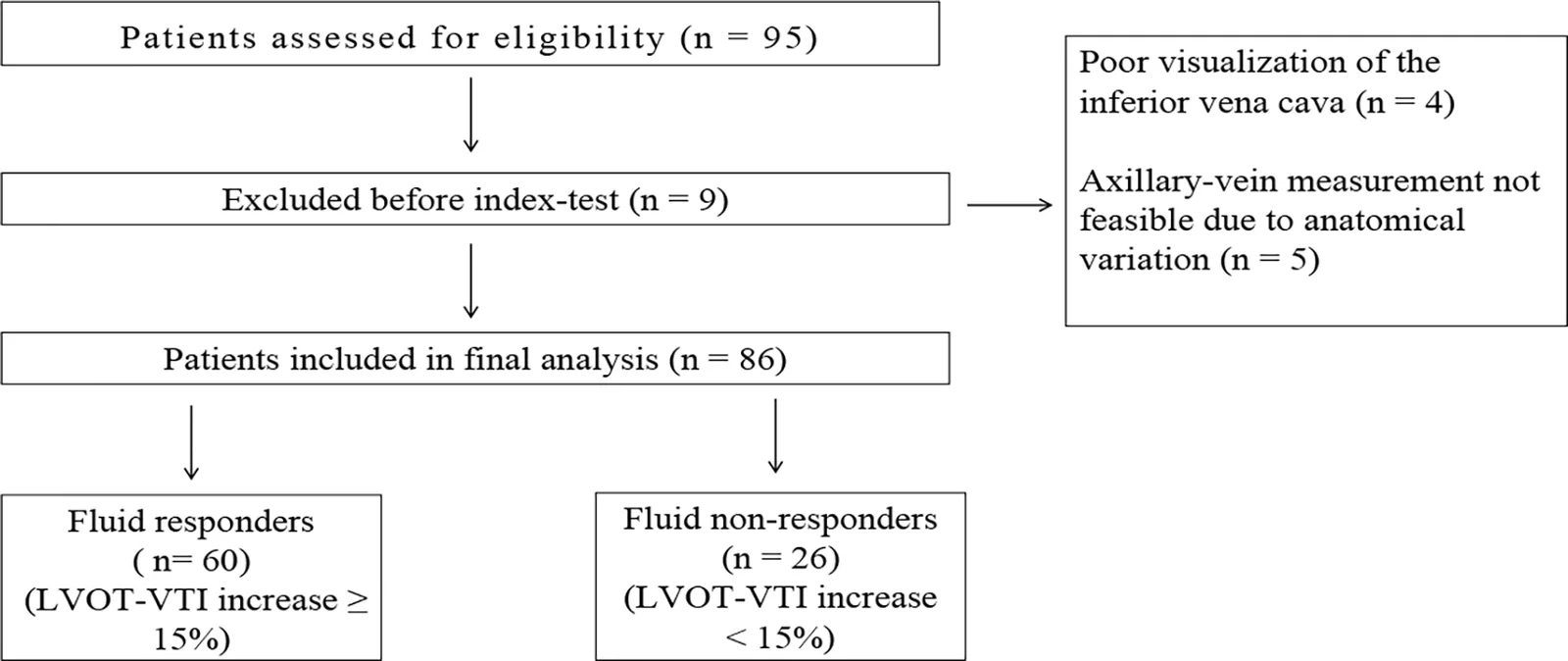
Flow diagram showing participant recruitment and grouping for this diagnostic-accuracy study, designed following the STARD 2015 recommendations.

3.2 No significant differences were observed between fluid responders and non-responders regarding gender distribution, primary infection sites, norepinephrine administration rate, APACHE-II score, height, body weight and BMI (all *P* > 0.05). There were significant differences in baseline age, SOFA score, serum lactate level, heart rate, mean arterial pressure (MAP) and central venous pressure (CVP) between the two groups (all *P* < 0.05). The fluid responder group had older age, higher baseline SOFA score, serum lactate level and heart rate, while the non-responder group showed higher baseline MAP and CVP (Table 1).

**Table 1:** Baseline demographic and clinical characteristics of all included patients.

Variables	Non-responders (*n* = 26)	Responders (*n* = 60)	*Z*/*χ*^2^	*P*-value
Demographic characteristics				
Age (years)	56 (42.00–70.00)	54.00 (35.00–67.00)	−2.439	0.015
Gender- *n* (%)			0.182	0.669
Male	18 (69.2)	38 (63.3)		
Female	8 (30.8)	22 (36.7)		
Height (m)	1.69 (1.62–1.72)	1.66 (1.60–1.72)	−0.259	0.796
Weight (kg)	70.97 (56.82–84.33)	71.22 (56.81–83.90)	−0.752	0.452
BMI	26.48 (19.57–28.17)	26.48 (20.13–28.17)	−1.315	0.189
Site of infection- *n* (%)			–	0.928
Respiratory	13 (50.0)	29 (48.3)		
Urinary	4 (15.4)	7 (11.7)		
Gastrointestinal	2 (7.7)	9 (15.0)		
Skin and soft tissue	3 (11.5)	4 (6.7)		
Abdominal	1 (3.8)	5 (8.3)		
Bloodstream	1 (3.8)	2 (3.3)		
Central nervous system	1 (3.8)	1 (1.7)		
Other	1 (3.8)	3 (5.0)		
Treatment-related parameter				
Norepinephrine use- (μg·kg⁻^1^·min⁻^1^)	0.5 (0.2–0.95)	0.7 (0.5–1.1)	−0.962	0.336
Clinical severity scores				
APACHE II	15.00 (10.00–19.00)	18.00 (12.00–23.00)	−1.153	0.126
SOFA	9.00 (7.00–11.00)	11.00 (8.00–12.00)	−2.157	0.031
Hemodynamic and metabolic parameters				
Lactate-(mmol/L)	4.30 (3.18–5.73)	5.45 (4.33–6.23)	−3.011	0.003
Heart rate (bpm)	78.00 (68.00–99.00)	99.00 (88.00–110.00)	−3.639	<0.001
MAP (mmHg)	68.00 (66.00–70.00)	62.00 (61.00–67.00)	−4.811	<0.001
CVP (cmH_2_O)	7.50 (6.88–8.10)	5.20 (4.90–6.90)	−4.233	<0.001

3.3 Compared with the fluid responder group, the fluid non-responder group had significantly higher baseline LVOT-VTI, dIVCmax and dAXVmax (all *P* < 0.05). In contrast, IVC-DI and AXV-DI in the non-responder group were markedly lower than those in the responder group, with all differences reaching statistical significance (*P* < 0.05). All ultrasonic hemodynamic parameters at baseline differed significantly between the two groups (all *P* < 0.05) (see Table 2).

**Table 2:** Baseline ultrasonic hemodynamic parameters of fluid responders and non-responders.

Parameters	Non-responders (*n* = 26)	Responders (*n* = 60)	*Z*-value	*P*-value
VTI (cm)	19.80 (18.92–21.78)	15.75 (15.30- 16.48)	−7.265	<0.001
dIVCmax (cm)	1.76 (1.69–1.81)	1.47 (1.32–1.53)	−5.552	<0.001
IVC-DI (%)	7.9 (6.04–14.04)	32.30 (17.86–35.62)	−6.314	<0.001
dAXVmax (cm)	0.68 (0.60–0.87)	0.58 (0.48–0.67)	−3.822	<0.001
AXV-DI (%)	17.66 (13.53–25.08)	32.23 (18.37–43.76)	−4.424	<0.001

3.4 We constructed the receiver operating characteristic (ROC) curves of IVC-DI and AXV-DI for predicting fluid responsiveness. The area under the curve (AUC) for IVC-DI was 0.93 (95% CI: 0.88–0.98), indicating excellent predictive performance. The AUC for AXV-DI was 0.80 (95% CI: 0.70–0.90), demonstrating good predictive ability. DeLong test revealed a statistically significant difference between the two AUC values (*P* = 0.009), confirming that the predictive performance of AXV-DI was inferior to IVC-DI. At the optimal cut-off value of 17.60 % (calculated via the Youden index), IVC-DI yielded a sensitivity of 0.78 (95% CI: 0.68–0.88) and a specificity of 0.96 (95% CI: 0.89–1.00). At the optimal cut-off value of 27.70 %, AXV-DI had a sensitivity of 0.65 (95% CI: 0.53–0.77) and a specificity of 0.85 (95% CI: 0.69–0.96). These results are illustrated in Figure 2 and Table 3.

**Figure 2:**
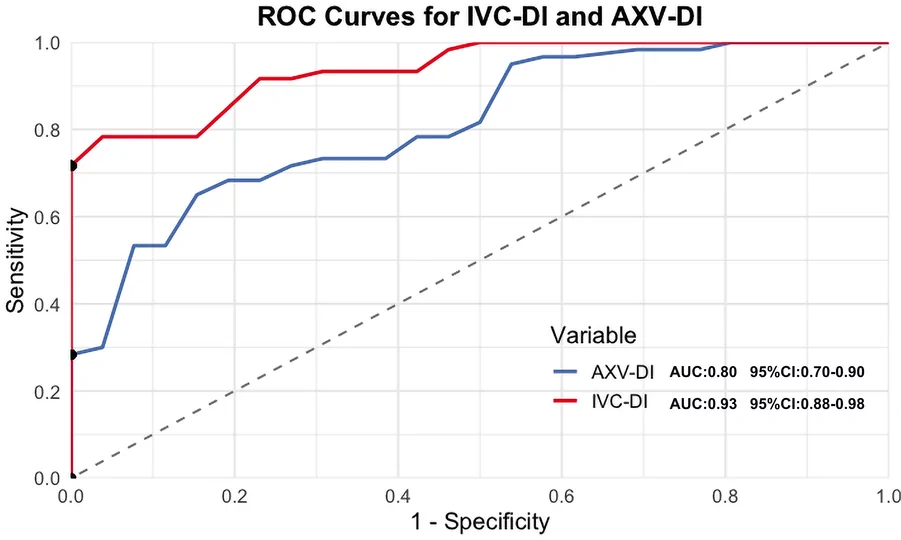
IVC-DI, inferior vena cava distensibility index. AXV-DI, axillary vein distensibility index.

**Table 3:** Diagnostic performance of IVC-DI and AXV-DI for predicting fluid responsiveness at their optimal cut-off values.

Index	Optimal cutoff	AUC (95% CI)	Sensitivity (95% CI)	Specificity (95% CI)	AUC comparison (DeLong)
IVC-DI (%)	17.60	0.93 (0.88–0.98)	0.78 (0.68–0.88)	0.96 (0.89–1.00)	*P* = 0.009
AXV-DI (%)	27.70	0.80 (0.70–0.90)	0.65 (0.53–0.77)	0.85 (0.69–0.96)	–

3.5 At baseline, significant between-group differences were observed in all measured indicators (all *P* < 0.05). Compared with responders, non-responders presented higher VTI, MAP, CVP, dIVCmax and dAXVmax, and lower, heart rate, IVC-DI and AXV-DI. These inter-group differences remained significant after the fluid challenge (all *P* < 0.05), with consistent trends consistent with baseline findings.

In the responder group, all parameters underwent significant alterations following fluid resuscitation (all *P* < 0.05). VTI, dIVCmax, dAXVmax, MAP and CVP were elevated, while heart rate, IVC-DI and AXV-DI were reduced. Individual-level changes in LVOT-VTI across the two time-points are shown in Figure 3. By contrast, only a portion of indicators changed significantly in the non-responder group (see Table 4).

**Figure 3:**
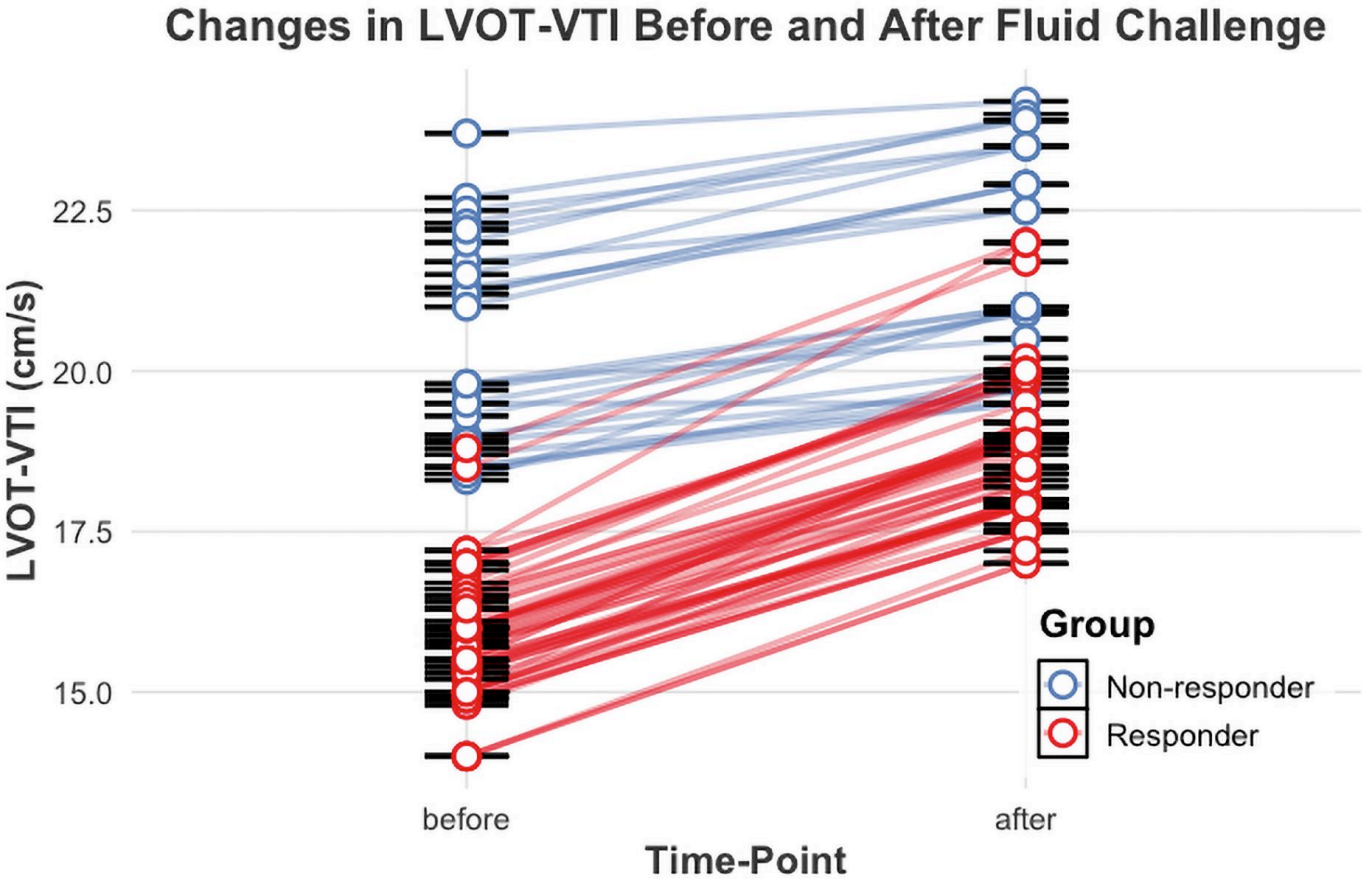
LVOT-VTI, Left ventricular outflow tract velocity-time integral. LVOT-VTI of individual patients before and after fluid challenge. LVOT-VTI increased markedly after fluid administration in both groups, with a greater magnitude of change in fluid responders.

**Table 4:** Hemodynamic parameters before and after fluid challenge in fluid responders and non-responders with septic shock.

Parameters	Time Point	non-responders (*n* = 26)	Responders (*n* = 60)	Inter-group Comparison (*Z*/*P*)	Intra-group Comparison (non-responders)	Intra-group Comparison (Responders)	Change-score Δ non-responder	Change-score Δ responder	Between-group comparison of Δ *(Z*/*P)*
VTI (cm)	Before	19.80 (18.92–21.78)	15.75 (15.30- 16.48)	−7.265/<0.001	−4.377/<0.001	−6.749/<0.001	–	–	–
	After	21.00 (19.98–23.50)	18.60 (17.92- 19.20)	−6.420/<0.001					
Heart Rate (bpm)	Before	78.00 (68.00–99.00)	99.00 (88.00–110.00)	−3.639/<0.001	−2.518/0.012	−4.670/<0.001	−5 (−20 to 0)	−8 (−14 to 0)	−0.179/0.858
	After	72.00 (66.00–84.00)	89.00 (79.00–99.00)	−4.742/<0.001					
MAP (mmHg)	Before	68.00 (66.00–70.00)	62.00 (61.00–67.00)	−4.881/<0.001	−1.428/0.153	−4.540/<0.001	0 (0–2)	1 (0–5)	−1.629/0.103
	After	69.00 (66.00–71.00)	66.00 (63.00–68.00)	−3.646/<0.001					
CVP (cmH_2_O)	Before	7.50 (6.90–8.10)	5.20 (4.90–6.90)	−4.233/<0.001	−3.558/<0.001	−5.701/<0.001	0.10 (0–0.2)	0.65 (0.05–1.70)	−3.781/<0.001
	After	7.90 (7.00–8.20)	6.90 (6.50–7.30)	−2.706/0.007					
dIVCmax (cm)	Before	1.76 (1.69–1.81)	1.47 (1.32–1.53)	−5.552/<0.001	−2.723/0.006	−5.508/<0.001	0 (0.01–0.03)	0.1 (0.02–0.16)	−4.279/<0.001
	After	1.78 (1.70–1.83)	1.58 (1.51–1.64)	−5.369/<0.001					
IVC-DI (%)	Before	7.9 (6.04–14.04)	32.30 (17.86–35.62)	−6.314/<0.001	−0.852/0.394	−5.674/<0.001	−0.11 (−1.52 to 0.75)	−10.21 (−19.14 to −0.43)	−4.236/<0.001
	After	7.93 (5.66–12.26)	18.22 (14.38–25.86)	−5.502/<0.001					
dAXVmax (cm)	Before	0.68 (0.60–0.87)	0.58 (0.48–0.67)	−3.822/<0.001	−2.124/0.034	−5.568/<0.001	0 (0–0.01)	0.045 (−0 to 0.1)	−4.201/<0.001
	After	0.68 (0.61–0.87)	0.65 (0.58–0.70)	−2.119/<0.034					
AXV-DI (%)	Before	17.66 (13.53–25.08)	32.23 (18.37–43.76)	−4.424/<0.001	−2.464/0.014	−3.645/<0.001	−2.29 (−11.5 to 0)	−5.78 (−16.61 to 0)	−0.951/0.342
	After	14.87 (10.08–17.40)	25.07 (14.51–34.50)	−3.959/<0.001					

Δ = value after fluid challenge minus baseline value. Change-score (Δ) represents the raw difference between value after fluid challenge and baseline value; negative values indicate a decrease, positive values indicate an increase. Between-group comparison of Δ was performed using the Mann–Whitney *U* test. The inter-group comparison (*Z*/*P*) column corresponds to cross-sectional between-group comparison at each single time-point. Intra-group comparisons refer to pre-post comparison within each group. No change-score comparison was performed for LVOT-VTI because responder status was defined based on the relative change of LVOT-VTI.

3.6 According to Figures 4–6 and Table 4, inter-group comparison at baseline showed that responders had significantly lower MAP, CVP, dIVCmax and dAXVmax, but higher IVC-DI and AXV-DI compared with non-responders (all *P* < 0.05). For within-group changes after fluid challenge, multiple hemodynamic and ultrasonic indices changed significantly in responders. In non-responders, significant intra-group changes were observed for HR, CVP, dIVCmax, dAXVmax and AXV-DI, while MAP (*P* = 0.153) and IVC-DI (*P* = 0.394) did not alter significantly. At post-fluid-challenge time-point, IVC-DI and AXV-DI remained higher in responders. Change-score analysis (Δ = value after fluid challenge minus baseline value, raw difference allowing positive and negative values) was applied to formally compare the magnitude of changes between groups. Significant between-group differences of Δ were detected for CVP, dIVCmax, IVC-DI and dAXVmax (all *P* < 0.001). In contrast, no inter-group differences in change magnitude were found for heart rate (*P* = 0.858), MAP (*P* = 0.103) and AXV-DI (*P* = 0.342). Change-score analysis was not performed for VTI to avoid circular reasoning, given that responder status was defined by the relative increment of VTI. All change-score results are presented in Table 4.

**Figure 4:**
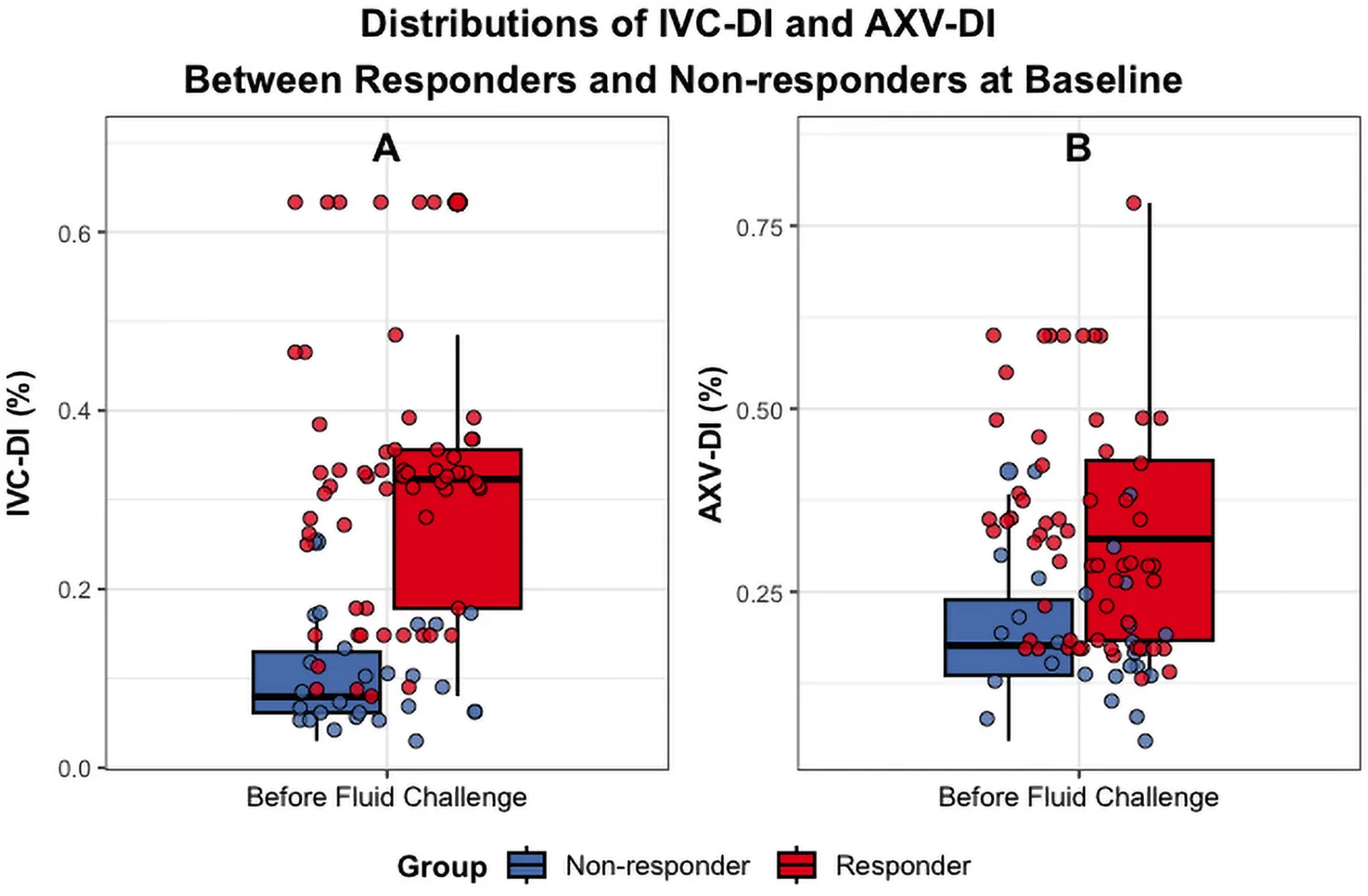
Box plots of baseline IVC_DI and AXV_DI in fluid responders and non_responders. Both indices were significantly higher in responders at baseline. (A) Baseline IVC_DI; (B) Baseline AXV_DI.

**Figure 5:**
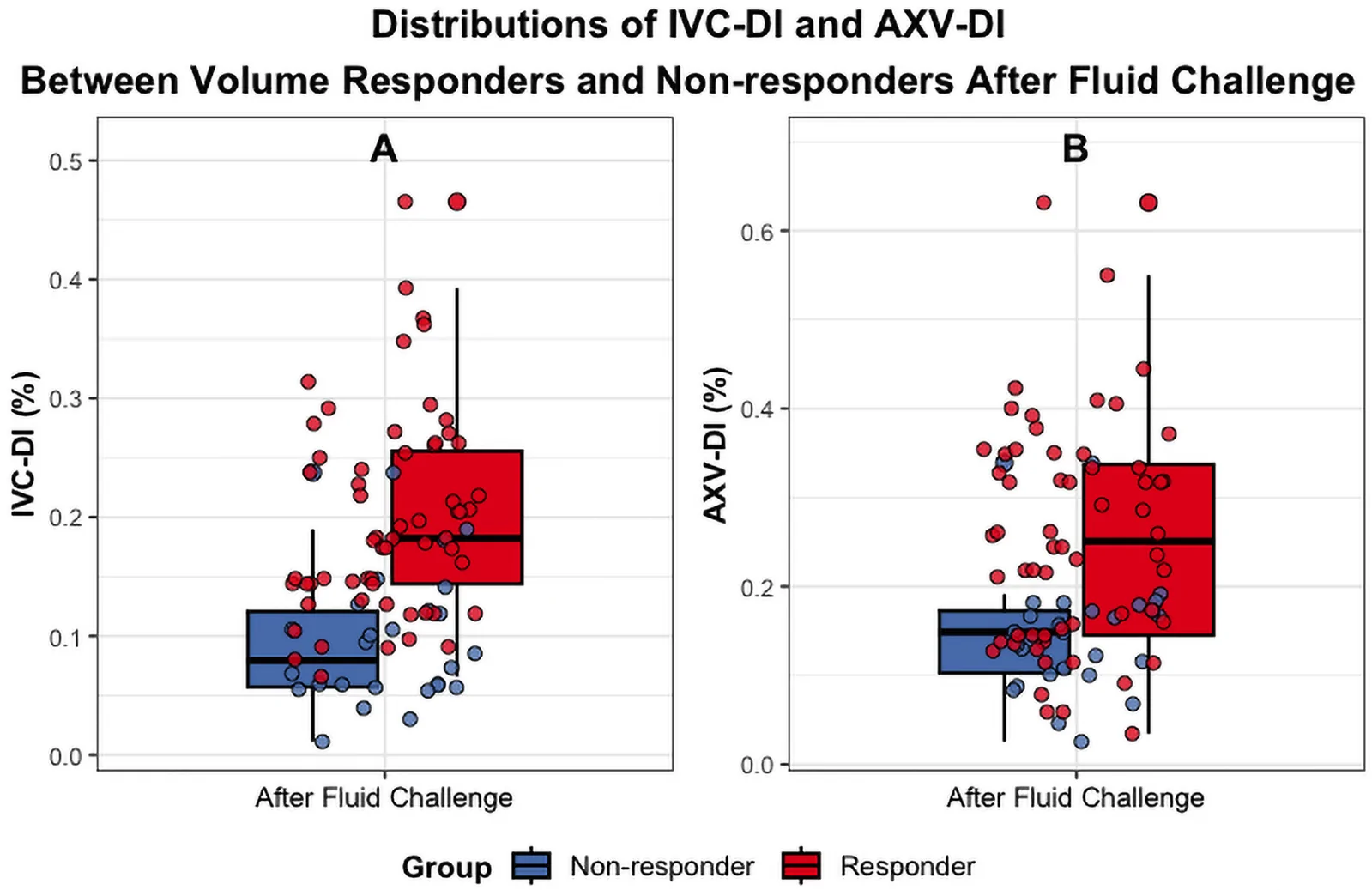
Box plots of IVC_DI and AXV_DI after fluid challenge. (A) Post_challenge IVC_DI; (B) Post_challenge AXV_DI.

**Figure 6:**
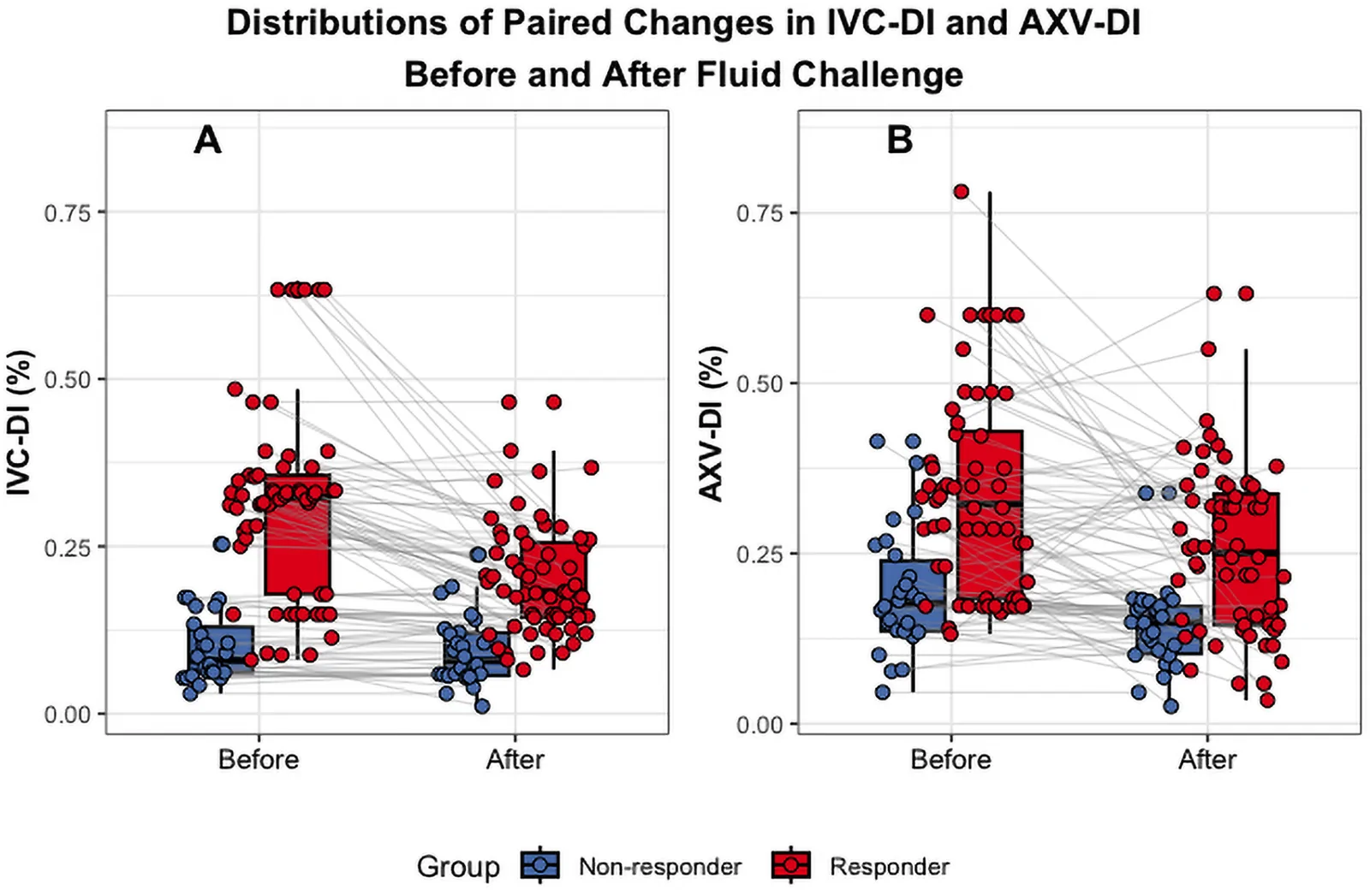
Dynamic changes of IVC_DI and AXV_DI before and after fluid challenge in the two groups. (A) Paired changes in IVC_DI; (B) Paired changes in AXV_DI.

## 4 Discussion

This prospective observational study showed that the respiratory variability of the axillary vein and inferior vena cava had predictive value for fluid responsiveness in mechanically ventilated patients with septic shock, although their predictive performance differed. We also analyzed changes in AXV-DI, IVC-DI and other hemodynamic parameters between fluid responders and non-responders. These indices exhibited distinct dynamic changes after fluid resuscitation and could provide auxiliary evidence for clinical volume assessment.

The primary outcome of this study focused on the head-to-head comparison of the diagnostic performance between AXV-DI and IVC-DI for predicting fluid responsiveness. In our cohort, the AUC of IVC-DI for predicting fluid responsiveness was 0.93, which was slightly higher than the pooled AUC of 0.88 reported in the meta-analysis by Zhang et al. (8). This discrepancy may be partly explained by our strict inclusion criteria: only patients with clearly visualized inferior vena cava images were enrolled in the present study, which might overestimate the diagnostic performance of IVC-DI. The DeLong test confirmed that this difference in diagnostic performance was statistically significant (*P* = 0.009).

Our data demonstrated that AXV-DI exhibited acceptable but inferior diagnostic performance relative to IVC-DI. Few prior head-to-head studies have assessed AXV-DI among patients with septic shock. Existing observational data regarding axillary-vein-derived indices were mainly obtained from postoperative or surgical-anesthesia populations, whose hemodynamic status differed from that of patients with septic shock. Apart from patient-population heterogeneity, positional confounders and the risk of probe-induced external compression on the distal axillary vein may further impair its predictive power in clinical practice.

The incremental value of the present study lies in that we performed a direct head-to-head comparison between AXV-DI and IVC-DI specifically in mechanically ventilated patients with septic shock. For the secondary outcome, we analyzed the dynamic changes of ultrasound-derived indices and hemodynamic parameters before and after standardized fluid challenge. Beyond their diagnostic performance for predicting fluid responsiveness, these ultrasound-derived indices displayed distinct dynamic patterns during fluid challenge between responders and non-responders. As illustrated in the paired individual plots, LVOT-VTI increased markedly in most responders following fluid loading, whereas minimal change was observed among non-responders. Consistently, both IVC-DI and AXV-DI decreased after fluid resuscitation in responders, reflecting diminished respiratory venous fluctuation after preload augmentation (19, 20). The paired-change diagrams further demonstrated synchronous dynamic shifts of AXV-DI alongside IVC-DI within individual patients, even though the baseline diagnostic accuracy of AXV-DI was inferior to IVC-DI. Although both groups exhibited reduced venous variability after fluid challenge, post-challenge IVC-DI and AXV-DI remained higher in responders relative to non-responders. Non-responders showed blunted hemodynamic responses overall, a finding consistent with limited preload reserve in these patients (21). From a clinical perspective, these dynamic changes reinforce that not only baseline values but also fluid-challenge-induced alterations of venous ultrasound indices may provide supplementary information for assessing volume status.

A recent meta-analysis focusing on septic patients reported that the pooled sensitivity, specificity and AUC of IVC-DI for predicting fluid responsiveness in mechanically ventilated septic patients were 0.79, 0.82 and 0.88 (95% CI 0.84–0.90), respectively (8). Meta-regression analysis indicated that studies applying a 500 mL fluid challenge tended to yield higher diagnostic accuracy than those using a 200 mL bolus, although this difference did not reach statistical significance (meta-regression *P* = 0.417). Considering this trend and the commonly adopted protocol in our ICU clinical practice, we chose a 500 mL fluid challenge in the present study. Notably, the optimal dIVC cut-off values reported across original studies varied widely from 10.2 % to 28 %, and no universally accepted threshold has been established to date.

Several limitations should be considered when interpreting our findings and extrapolating conclusions to broader clinical populations. First, patients with absent or poor–quality inferior vena cava ultrasound images were excluded, which may introduce selection bias and potentially overestimate the diagnostic accuracy of IVC–DI. Accordingly, the potential value of AXV-DI as an alternative tool in patients with suboptimal IVC visualization could not be verified in the current cohort. Second, all ultrasound examinations were performed by a single experienced operator, and intra- and inter-observer reproducibility was not quantitatively assessed in the study protocol, with no available intraclass correlation coefficient data. Image acquisition variability may affect the measurements of LVOT-VTI, IVC-DI and AXV-DI, and could further influence the 15% VTI variation threshold for defining fluid responsiveness, although serial volume-oriented ultrasound assessments are routinely performed in clinical practice. Third, this exploratory single-center diagnostic study had a relatively small sample size, with no *a priori* power calculation for AUC comparison. Given the limited sample, wide confidence intervals and absence of external validation, our findings are preliminary and should be interpreted cautiously. Fourth, we enrolled only fully sedated, paralyzed, mechanically ventilated adults with septic shock, excluding patients with spontaneous breathing, arrhythmia, severe right heart dysfunction, or pregnancy. The strict inclusion criteria maintained good internal validity but limited external generalizability; thus, our conclusions cannot be directly extrapolated to patients with spontaneous breathing or severe underlying cardiac disease. These limitations motivate our ongoing data collection and larger subsequent study, aiming to validate AXV-DI and IVC-DI across diverse clinical settings and extend our findings to a broader population of critically ill patients.

In summary, both AXV-DI and IVC-DI can predict fluid responsiveness in mechanically ventilated patients with septic shock. However, AXV-DI exhibits lower diagnostic performance than IVC-DI.

## Data Availability

The original contributions presented in the study are included in the article/Supplementary Material, further inquiries can be directed to the corresponding author.
